# Deciphering the genetic basis of novel traits that discriminate useful and non‐useful biomass to enhance harvest index in wheat

**DOI:** 10.1002/tpg2.20512

**Published:** 2024-09-18

**Authors:** Dipendra Shahi, Jia Guo, Md Ali Babar, Sumit Pradhan, Muhsin Avci, Naeem Khan, Jordan McBreen, Smita Rayamajhi, Zhao Liu, Guihua Bai, Paul St. Amand, Amy Bernardo, Matthew Reynolds, Gemma Molero, Sivakumar Sukumaran, John Foulkes, Jahangir Khan

**Affiliations:** ^1^ School of Plant, Environmental and Soil Sciences Louisiana State Agricultural Center Baton Rouge Louisiana USA; ^2^ Inari Agriculture West Lafayette Indiana USA; ^3^ Department of Agronomy University of Florida Gainesville Florida USA; ^4^ Department of Agronomy Kansas State University Manhattan Kansas USA; ^5^ USDA‐ARS, Hard Winter Wheat Genetics Research Unit Manhattan Kansas USA; ^6^ CIMMYT International Maize and Wheat Improvement Center (CIMMYT) El Batan Texcoco Mexico; ^7^ Plant & Environmental Sciences & Advanced Plant Technology Program Clemson University Clemson South Carolina USA; ^8^ Division of Plant and Crop Sciences, School of Biosciences University of Nottingham Leicestershire UK; ^9^ PARC‐Balochistan Agricultural Research and Development Center Quetta Pakistan

## Abstract

Wheat (*Triticum aestivum* L.) production must be doubled in the next 25 years to meet the global food demand. Harvest index (HI) is an important indicator of efficient partitioning of photosynthetic assimilates to grains. Reducing competition from alternative sinks, such as stems, and deviating assimilates toward grain increase grain number (GN), HI, and grain yield (GY). Novel partitioning traits have great potential to be utilized in wheat breeding programs to increase HI. In this study, we evaluated 236 US facultative soft wheat genotypes for multiple stem and spike partitioning traits at 7 days after anthesis, and for GN, HI, and GY in two locations of Florida in 2016–2017 and 2017–2018 wheat growing seasons. The panel was genotyped with 20,706 single nucleotide polymorphisms generated by genotype‐by‐sequencing approach. Spike partitioning index (SPI) showed negative significant correlations with lamina partitioning index and true stem partitioning index. Internode 2 and 3 lengths and partitioning indices had significant negative correlations with SPI and HI. The results indicate enhanced competition for assimilates between spikes and second and third internodes during stem elongation. Genome‐wide association study (GWAS) identified 114 unique significant marker‐trait associations (MTAs) for 12 traits, and 58 MTAs were found within genes that encode different proteins related to biotic/abiotic stress tolerance and other functions. Significant MTAs identified in the GWAS were converted into kompetitive allele specific PCR markers. Some of the markers were validated and can be effectively employed in marker‐assisted selection to improve HI, GY, and GN.

## Core Ideas

- Genome‐wide association study was conducted for 12 harvest index related traits using 236 wheat genotypes.
- Significant marker‐trait associations (MTAs) were found and the kompetitive allele specific PCR markers were developed and validated for some of the MTAs.
- These markers associated with harvest index related traits can be used in marker‐assisted selection.

**Abbreviations**
ANOVA: analysis of variance
BLUE: best linear unbiased estimate
DM: dry matter
GN: grain number
GWAS: genome‐wide association study
GY: grain yield
HI: harvest index
I2L: internode 2 length
I2PI: internode 2 partitioning index
I3L: internode 3 length
I3PI: internode 3 partitioning index
KASP: kompetitive allele specific PCR
LD: linkage disequilibrium
LPI: lamina partitioning index
MTA: marker‐trait association
PC: principal component
PVE: phenotypic variation explained
QTL: quantitative trait locus
SPI: spike partitioning index
TSPI: true stem partitioning index

## 1 INTRODUCTION

Wheat (*Triticum aestivum* L.) is one of the major staple food crops for global food security, providing 20% of global calorie and protein requirements (Ding et al., 2023; Nouraei et al., 2024). There is a rising food demand due to the increasing global population, but the situation has been complicated by climate change, land degradation, and declining natural resources. Genetic gain in wheat is lower than 1% per annum (M. Reynolds et al., 2012; Tadesse et al., 2019; Tessema et al., 2020), although an annual increase of 2%–3% is expected to meet the projected food demand by the year 2050 (Crespo‐Herrera et al., 2017; Hawkesford et al., 2013). Harvest index (HI, grain yield [GY]/aboveground biomass) is a widely used criterion for maximizing GY. It is also an indicator of efficient partitioning of assimilates to grains in plants. Although researchers have suggested that HI value can reach up to 65% in wheat (Foulkes et al., 2011), it has remained largely unchanged for a few decades and is typically less than 50% for spring wheat and 50%–55% for winter wheat under irrigated conditions (Foulkes et al., 2011; Pradhan et al., 2019; Shearman et al., 2005). A significant change (∼20%) in yield potential could be achieved through a stable expression of HI at values of 55% and above. However, researchers have a limited understanding of the genetic basis of different stem and spike partitioning traits that could effectively increase HI and thus ultimately improve GY potential in wheat (Foulkes et al., 2011; Pradhan et al., 2019).

Modern wheat cultivars have displayed an increase in biomass mostly due to enhanced radiation‐use efficiency and photosynthesis (Aisawi et al., 2015; Beche et al., 2014; Donmez et al., 2001; Foulkes et al., 2011; Shearman et al., 2005). An increase in biomass has demonstrated a moderately positive association with GY (Pradhan et al., 2019; Shearman et al., 2005; Singh et al., 1998). Its negative association with HI in CIMMYT spring wheat (Aisawi et al., 2015) indicated a lower efficiency of modern cultivars (with high biomass) to mobilize assimilates toward grains. For a noteworthy change in HI and yield, we will need to identify traits that discriminate total biomass into “useful” and “non‐useful” biomass (Foulkes et al., 2011; Rivera‐Amado et al., 2019). Allocating extra biomass (obtained through increased photosynthesis) toward useful structures to maximize grain number (GN) and weight, instead of “non‐useful” biomass, would be an effective strategy to make significant genetic progress in HI in wheat. There is also a need to develop genomic tools associated with the traits that favor partitioning to “useful” rather than “non‐useful” biomass to maximize HI expression.

Various researchers have shown that increasing the GN is an effective way for genetic improvement of HI and yield potential (Foulkes et al., 2011; Pradhan et al., 2019). In recent years, spike partitioning index (SPI) (spike dry matter [DM]/aboveground DM at anthesis), along with fruiting efficiency, has emerged as a key candidate trait for improving GN by making more of the total assimilates available to reproductive structures (Gaju et al., 2009, 2014; M. Reynolds et al., 2012). SPI has been positively associated with grains m^−2^ and HI (Ferrante et al., 2010, 2013; Rivera‐Amado et al., 2019), and the presence of a wide range of variation for SPI indicates the wide scope for selection of this trait to improve HI and GN (Lázaro & Abbate, 2012; Rivera‐Amado et al., 2019). Rapid spike growth period of the stem elongation phase is reported to be a critical time for GN determination in wheat. Since the growth of stem and spike overlap during this phase, these plant parts compete vigorously with each other for assimilates. Diverting more assimilates toward the growing spike during this phase from booting to anthesis has been shown to increase floret survival, thereby increasing spike fertility and GN (Fischer, 2007; Foulkes et al., 2011; Gaju et al., 2009; González et al., 2011; Miralles & Slafer, 2007). Partitioning of biomass to the spike during this phase can be increased by reducing the DM partitioning toward other competing plant parts such as stem (Fischer, 2007; Foulkes et al., 2009, 2011; M. P. Reynolds et al., 1999; M. Reynolds et al., 2009).

Evidence showed that the structural stem competes most strongly with the spike for assimilates during the stem elongation period and reduction of DM in structural stem can increase spike dry weight and GN at anthesis (Beed et al., 2007; Slafer, 2003). Other studies suggest that high stem soluble DM is favorable for floret survival and grains m^−2^ (Dreccer et al., 2009). Researchers have suggested that improvement in HI can be driven through (1) increased spike DM partitioning by anthesis and (2) decreased stem DM partitioning while providing sufficient structural strength and increased partitioning to the soluble fraction of the stem (i.e., carbohydrate reserves) (Aisawi et al., 2015; Foulkes et al., 2009; M. Reynolds et al., 2012). Competition between spike and stem for plant biomass is also indicated through a negative association between the SPI and the stem partitioning index (Foulkes et al., 2011; Rivera‐Amado et al., 2019). Studies of partitioning indices of the spike and other plant parts will help understand the assimilate partitioning process in wheat. As the genetic basis of these partitioning traits is not clearly understood (M. Reynolds et al., 2012), identifying genetic loci controlling these partitioning traits would be critical for the genetic progress of HI and GY (Rivera‐Amado et al., 2019; Sierra‐Gonzalez et al., 2021).

Genome‐wide association studies (GWASs) are a powerful approach that uses historical recombination and linkage disequilibrium (LD) to dissect the genetic basis of complex traits (F. Li et al., 2019). In recent years, GWAS has also been utilized extensively in wheat to understand the genetic architecture of different quantitative traits. However, there has been very little research regarding stem and spike partitioning traits in wheat. GWAS on these partitioning traits can be an important step in identifying and employing molecular markers linked to quantitative trait loci (QTLs) for indirect selection in wheat breeding programs. The objectives of this study were to (i) evaluate the association of stem and spike partitioning traits at 7 days after anthesis with HI, GY, and GN in high biomass yielding wheat background, (ii) identify QTLs and molecular markers associated with these partitioning traits that permit photosynthetic products to be consistently translated to higher HI through GWAS analysis, (iii) identify traits and markers that enable discrimination between “useful” and “non‐useful” biomass to improve partitioning towards the grain, and (iv) build kompetitive allele specific PCR (KASP) markers based on the identified single nucleotide polymorphisms (SNPs) and validate them in a new population.

**Core Ideas**

- Genome‐wide association study was conducted for 12 harvest index related traits using 236 wheat genotypes.
- Significant marker‐trait associations (MTAs) were found and the kompetitive allele specific PCR markers were developed and validated for some of the MTAs.
- These markers associated with harvest index related traits can be used in marker‐assisted selection.

## 2 MATERIALS AND METHODS

### 2.1 Genetic materials and experimental design

We evaluated an association panel of 236 facultative soft wheat elite lines and varieties adapted to south and southeastern United States’ soft wheat‐growing regions. The genotypes and environments used in this study have been described previously (Shahi et al., 2022). In brief, the research was conducted at the Plant Science Research and Education Center in Citra, FL, in 2017–2018, and North Florida Research and Education Center in Quincy, FL, for 2 years, 2016–2017 and 2017–2018. Each experiment was planted in a 5.1 m^2^ plot (3.33‐m long/1.52‐m wide) using a modified augmented design with three repeated checks (SS8641, PI 674197; AGS2000, PI 656845; Jamestown, PI 653731) at a seed rate of 100 kg ha^−1^ and six rows per plot. The experiments were planted between November 15 and 20 in both locations. The three repeated checks were widely adapted and cultivated throughout the southeastern United States. The checks were replicated twice in each block, comprising about 16% of the total plots. Management practices such as fertilizer, chemical application, and irrigation were used as recommended for best management practices for proper growth and yield. To control foliar and glume diseases, fungicides were sprayed two to three times. The average monthly temperature dipped from November to December, with temperatures reaching their lowest point in January and starting to increase again up to April/May for all environments. The weather data for two locations over two growing seasons are shown in Figure S1.

A validation panel consists of two diversity populations including 178 facultative and 59 spring wheat lines collected from University of Idaho, University of Arkansas, Virginia Tech, University of Georgia, North Carolina State University, and Louisiana State University. The panel was evaluated in the 2018–2019 season using a randomized augmented design with three repeated checks (AGS 2000, SS8641, and Jamestown). Each line was planted in six‐row plots (3 m × 1.5 m) at the seeding rate of 100 kg h^−1^. Pesticides were sprayed to control local diseases, weeds, and insects as needed. Fertilizer and irrigation were applied based on plant growth stages and field moisture conditions to avoid any water or nutrient deficiency, respectively.

### 2.2 Phenotyping

For the GWAS studies, 12 phenotypic traits including HI, GY, GN, plant height (Ht), and biomass partitioning traits were collected in the present study. Days to anthesis was taken for each plot as the days from planting at which 50% of plants flowered (Zadoks et al., 1974). At 7 days after anthesis (Zadoks scale: GS70), a plant sample was cut at ground level from a 0.25 m^2^ area of each plot. The sample was oven‐dried at 60°C for 72 h and dried biomass weight at anthesis plus 7 days (BM [A + 7d]) was taken and converted to g m^−2^. For DM partitioning (spike, leaf lamina, and stem), 10 randomly selected fertile shoots (those with a spike) from the dried sample were separated into spike, leaf sheath, leaf lamina, and true stem (devoid of leaf lamina and leaf sheath) and their weight was taken. The partitioning indices of each component (SPI, leaf lamina partitioning index [LPI] (Shahi et al., 2022), and true stem partitioning index [TSPI]) were calculated as the ratio of each component DM to aboveground DM. For example, SPI was calculated as a ratio of total spike DM and aboveground DM. Each true stem was further cut into internodes; weight and lengths of internode 2 (I2L) and internode 3 (I3L) were taken (internode 2 being the internode below the peduncle and peduncle was considered as internode 1). The partitioning index of each internode was estimated as a ratio of internode DM to aboveground DM (internode 2 partitioning index [I2PI] = internode 2 DM/aboveground DM: internode 3 partitioning index [I3PI] = internode 3 DM/aboveground DM).

Data for HI, GY, GN, and Ht were recorded at physiological maturity (Zadoks scale: GS90). Days to physiological maturity for each plot was taken when 50% peduncle turned yellow. For each plot, Ht was measured in four randomly selected plants from the soil surface to the tip of the spike excluding awns and was expressed in centimeter as an average of all measurements. At physiological maturity, tillers from 0.25 m^2^ plot area were harvested and the spike number was counted and converted to spikes m^−2^. HI was measured as the ratio of GY m^−2^ to total dry biomass m^−2^. Note that 1000‐grain weight (TGW) was calculated by counting 1000 kernels by a seed counter and weighing them. GN was obtained by dividing GY by individual grain weight (TGW/1000). Likewise, GY was measured in kg ha^−1^ as a total weight of machine‐harvested GY m^−2^ adjusted to 12% moisture content. The same set of phenotypic traits were measured in the validation studies except for plant Ht.

### 2.3 SNP genotyping, LD, population structure, and KASP marker development

DNA was extracted from fresh young leaves collected at three leaf stage using a modified cetryltrimethylammonium bromide protocol (Saghai‐Maroof et al., 1984). Genotyping‐by‐sequencing (GBS) was performed using two restriction enzymes *MspI* and *PstI‐HF* (Poland & Rife, 2012). The libraries were pooled in 96‐plex and sequenced in an Ion Torrent Proton sequencer (ThermoFisher Scientific) at the USDA Central Small Grain Genotyping Lab, Manhattan, KS.

The IWGSC reference genome RefSeq v1.0 (Appels et al., 2018) was used to align GBS reads with Burrows–Wheeler aligner program. SNPs were called using TASSEL v5.0 GBS v2.0 discovery pipeline (Bradbury et al., 2007) and aligned to the IWGSC reference genome RefSeq v1.0 (Appels et al., 2018) Markers obtained were filtered by keeping those SNPs with minor allele frequency (MAF) >5% and missing data <20%. After imputation with LD‐KNNi method implemented in TASSEL v.5, the imputed data were refiltered to remove those SNPs with MAF <5% or heterozygous call frequency <10%.

LD analysis was performed across A, B, D, and combined genomes using TASSEL v.5 to estimate LD values (*r* ^2^) between pairs of SNP markers along each chromosome. To visualize the LD decay pattern, LD values (*r* ^2^) were plotted against their physical distance (Mbp). The threshold value of the LD (*r* ^2^) was set at 0.1, which is a commonly applied value in many related studies (Sehgal et al., 2020; Zhu et al., 2008). Locally weighed polynomial regression (LOESS) curves were then fitted into the scatter plot. The point at which the LOESS curve intercepts the threshold *r* ^2^ (0.1) was determined as the average LD decay of the genome. The population structure of the association panel was determined by using discriminant analysis of principal components (DAPC) using R package “adegenet” (Jombart et al., 2010). The best number of clusters (genetic groups) was inferred using the Bayesian information criterion score provided by DAPC.

The significant SNPs from GWAS were selected for KASP marker development. KASP primers were designed using PolyMarker (http://www.polymarker.info/) based on the sequences flanking the SNPs in Chinese Spring reference genome RefSeq 1.0 (IWGSC 2018). The primers were then used for genotyping of the population. The KASP assays were performed in a GeneAmp PCR System 9700 Fast Thermal Cycler using a 5 µL reaction mix consisting of 1.94 µL of 2 × PACE Genotyping Master Mix (https://3crbio.com/), 0.06 µL primer mix, and 3 µL DNA at 10–20 ng µL^−1^. The PCR profile started with denaturation of 94°C for 15 min, followed by 10 touch‐down PCR cycles at 94°C for 20 s and 67°C for 60 s with −1.0°C/cycle, and then 32 cycles at 94°C for 20 s and 57°C for 60 s. KASP marker assays were conducted at USDA‐ARS Central Small Grain Genotyping Lab, Manhattan, KS.

### 2.4 Phenotypic data analysis

Analysis of variance (ANOVA) was conducted using the “lme4” package (Bates et al., 2015) in R (v3.5.1, R Development Core Team). The best linear unbiased estimates (BLUEs) were obtained for individual environments and combined environments. All traits were adjusted using days to anthesis as a covariate. When estimating BLUEs for individual environments, the following model was used:

$$Y_{ijkl}=\mu +B_{i}+ID_{j}+G_{k}+C_{l}+\varepsilon_{ijkl}$$

For combined environments, the following model was used:

$$\begin{array}{ccc} Y_{ijklm} & = & \mu +ID_{j}+G_{k}+C_{l}+E_{m\;}+ID_{j}\times E_{m\;}+G_{k}\times E_{m\;}\\ & & +\;\;C_{l}\times E_{m\;}+B_{i}(E_{m)\;}+\varepsilon_{ijklm} \end{array}$$

where *Y* is the phenotype of a trait of interest, *μ* is the effect of the mean, *B_i_* is the effect of *i*th block, *G_k_* is the effect of *k*th genotypes, *C_l_* is the effect of the *l*th checks on each block, *E_m_* is the effect of the *m*th environment, and ID*_j_* is the effect of *j*th IDCheck. IDCheck was used to differentiate the effects of one check over the other checks, as well as the number of checks present on each block; ID*_j_* × *E_m_*, *G_k_* × *E_m_*, and *C_l_* × *E_m_* are the effects of check identifier by environment, genotype by environment, and check by environment interactions, respectively. *B_i_* (*E_m_*) is the effect of *i*th block nested within *m*th environment and *ε* is the residual.

Broad‐sense heritability was calculated assuming genotype and other effects as random (Shahi et al., 2022) and was obtained by:

$$H^{2}=\frac{\sigma^{2}G}{\sigma^{2}G+\frac{{\sigma^{2}}_{G\times E}}{n}+\frac{\sigma^{2}e}{nr}}\;$$

where *H* ^2^ is the broad‐sense heritability estimate, $\sigma^{2}G$ is the genetic variance, ${\sigma^{2}}_{\mathrm{GXE}}$ is the genotype by environmental variance, $\sigma^{2}e$ is the residual variance; *n* is the number of environments, and *r* is the number of replications.

Pearson's correlation among traits was calculated from BLUEs in R using the “corrplot” package in R (Wei, 2017). Principal component (PC) biplots were generated in R by using the “factoextra” package (Kassambara et al., 2017). Path coefficient analysis was also carried out in R using “lavaan” package (Rosseel, 2012) to estimate direct and indirect effects of different traits on HI. Traits such as GN, BM (A + 7d), SPI, LPI, TSPI, I2PI, I3PI, I2L, I3L, and Ht were used as predictors, whereas HI was designated as a response.

### 2.5 GWAS, haplotype block analysis, gene annotation, and validation analysis

A GWAS was conducted in Genome Association Predicted Integrated Tool in R (Lipka et al., 2012) using the compressed mixed linear model where population structure and kinship were used as cofactors and individuals are compressed into groups to optimize the computation and accuracy. GWAS was performed using BLUEs from Citra (2017–2018) (C18) and Quincy (2016–2017) (Q17), Quincy (2017–2018) (Q18), and combined datasets. A threshold of false discovery rate (FDR) of 10% (pFDR < 0.10) (Benjamini & Hochberg, 1995) was used to determine significant marker‐trait associations (MTAs). R package “cmplot” was used to construct SNP density, Manhattan, and *Q*–*Q* plots for visualization. Haplotype blocks of significant MTAs, which could be indicative of potential QTL regions, were visualized using “LDheatmap” package in R (Shin et al., 2006). Significant MTAs, which were common for two or more traits, were distinguished as MTAs with pleiotropic effect. Prospective genes associated with significant MTAs and their annotation were determined using IWGSC reference genome (RefSeq v1.0) (Appels et al., 2018) using Variant Effect Predictor tool in ensemble plant website (http://plants.ensembl.org/index.html).

In the validation study, genotypes were grouped by marker alleles and a student's *t*‐test was performed using BLUE values for comparison of group means. A KASP marker was considered an effective marker when the mean difference between two genotypic groups with contrasting marker alleles was significant. Kruskal–Wallis test was used if non‐normal residual was observed. Differences in means between lines with two or combinations of allele types were also calculated.

## 3 RESULTS

### 3.1 Genetic variation and broad‐sense heritability

A combined ANOVA showed significant genotypic variation for all the measured traits (Table S1). Environments and genotype‐by‐environment interaction demonstrated significant effects on all traits as well. The mean values and heritability of BM(A + 7d), I2L, I3L, LPI, TSPI, I2PI, I3PI, and Ht are listed in Table 1. The mean values ranged from 3182 g m^−2^ (Q17) to 3994 g m^−2^ (C18) for BM(A+7d), and 70.9 cm (C18) to 90.5 cm (Q18) for Ht. For stem partitioning traits, the mean values ranged from 0.42 (Combined, C18, and Q17) to 0.48 (Q18) for TSPI, from 0.12 (Q17) to 0.14 (C18 and Combined) for LPI, from 0.12 (Q17 and Combined) to 0.14 (Q18) for I2PI, and from 0.09 (C18) to 0.12 (Q18) for I3PI. For I2L and I3L, the mean phenotypic values ranged from 16.01 cm (C18) to 21.29 cm (Q18) and 10.33 cm (C18) to 15.02 cm (Q18), respectively.

**TABLE 1:** Summary of adjusted means and broad‐sense heritability (*H* ^2^) of phenotypic traits evaluated in the combined analysis.

Traits	C18	Q17	Q18	Combined	*H* ^2^
BM(A+7d)	3994	3182	3940	3231	0.13
LPI	0.14	0.12	0.13	0.14	0.20
TSPI	0.42	0.42	0.48	0.42	0.24
I2PI	0.13	0.12	0.14	0.12	0.27
I3PI	0.09	0.10	0.12	0.10	0.23
I2L	16.01	17.49	21.29	17.29	0.27
I3L	10.33	12.29	15.02	12.28	0.30
Ht	70.9	85.8	90.5	80.0	0.64

*Note*: C18, Q17, Q18, and Combined refers to Citra 2018, Quincy 2017, Quincy 2018, and combined datasets, respectively.

Abbreviations: BM (A+7d), above‐ground biomass at anthesis+7 days in g m^−2^; Ht, plant height in cm; I2L, internode 2 length in cm; I2PI, internode 2 partitioning index; I3L, internode 3 length in cm; I3PI, internode 3 partitioning index; LPI, lamina partitioning index; TSPI, true stem partitioning index.

The phenotypic values for HI, GY, GN, and SPI were reported before by Shahi et al. (2022). In summary, the mean values were ranged from 0.42 (C18) to 0.46 (Q17) for HI, from 4483 kg ha^−1^ (C18) to 5047 kg ha^−1^ (Q18) for GY, from 8012 m^−2^ (Q17) to 13,212 m^−2^ (Q18) for GN, and from 0.28 (C18) to 0.34 (Q18) for SPI (Shahi et al., 2022).

Broad‐sense heritability (*H* ^2^) varied for different traits (Table 1). The highest heritability was recorded for Ht (0.64), whereas the lowest heritability was estimated for BM(A+7d) (0.13) and LPI (0.20). The heritability of major partitioning traits was medium low from 0.23 to 0.30.

### 3.2 Phenotypic correlation and path coefficient analysis

HI showed positive correlations with GY (0.50** to 0.63**), GN (0.42*to 0.50**), and SPI (0.26** to 0.40**) in our previous paper (Shahi et al., 2022), whereas it had negative correlations with TSPI (−0.13* to −0.32**), I2PI (−0.14* to −0.22**), I3PI (−0.15* to −0.51**), I2L (−0.17* to −0.40**), and I3L (−0.27 ** to −0.34**) (Table 2). HI had no significant association with BM (A+7) in any environments tested in this study. Likewise, GY had significant negative correlations with I2PI (−0.1 to 0.23**) and I3PI (−0.08 to −0.25**). SPI demonstrated negative correlations with LPI (−0.16* to −0.55**), TSPI (−0.39**to −0.65**), I2PI (−0.20**to −0.34**), I3PI (−0.18* to −0.25**), I2L (−0.20** to −0.46**), and I3L (−0.24** to −0.38**). TSPI was positively correlated with I2PI, I3PI, I2L, and I3L. I2PI and I3PI had significant correlations with I2L (0.24** to 0.33**) and I3L (0.25** to 0.54**), respectively. I2L and I3L also demonstrated a significant positive correlation with plant height. Among 10 traits used as predictors for path coefficient analysis, GN (0.391**) and SPI (0.102*) had a positive direct effect, whereas LPI (−0.125*), I2L (−0.004), I3L (−0.122*), and I3PI (−0.107) had negative direct effects on HI in combined dataset (Table 3). The indirect effects of GN via SPI (0.024) and I3PI (0.012) were positive. Stem partitioning component traits (I2PI and I3PI) demonstrated negative indirect effects on HI via SPI and GN (Table 3). Path coefficient analysis of other datasets has been provided in Table S2.

**TABLE 2:** Pearson's correlation coefficients among phenotypic traits using best linear unbiased estimates (BLUEs) evaluated in three field experiments.

C18	HI	GY	GN	BM(A+7d)	SPI	LPI	TSPI	I2PI	I3PI	I2L	I3L	Ht
**BM(A+7d)**	−0.06	−0.02	0.05	1								
**LPI**	0.02	0	−0.02	−0.33**	−0.37**	1						
**TSPI**	−0.21*	−0.06	−0.11	0.13	−0.47**	0.06	1					
**I2PI**	−0.14*	−0.1	−0.04	−0.22**	−0.23**	0.09	0.24**	1				
**I3PI**	−0.15*	−0.08	−0.05	−0.24**	−0.23**	0.11	0.22*	0.72**	1			
**I2L**	−0.31**	−0.12	−0.17*	0.07	−0.20**	−0.02	0.31**	0.33**	0.28**	1		
**I3L**	−0.34**	−0.09	−0.15	0.04	−0.32**	0.07	0.19*	0.22**	0.37**	0.41**	1	
**Ht**	0	0.1	0.09	0.36**	−0.27**	−0.05	0.41**	−0.12	−0.12	0.16*	0.18*	1

Q17	HI	GY	GN	BM(A+7d)	SPI	LPI	TSPI	I2PI	I3PI	I2L	I3L	Ht
**BM(A+7d)**	0.06	0.09	0.02	1								
**LPI**	0.03	0.14*	0.15*	0.26**	−0.55**	1						
**TSPI**	−0.25**	−0.01	0	−0.05	−0.59**	0.05	1					
**I2PI**	−0.22**	−0.23**	−0.29**	−0.16*	−0.20**	−0.03	0.18*	1				
**I3PI**	−0.34**	−0.25**	−0.19**	−0.20**	−0.18*	−0.17*	0.26**	0.53**	1			
**I2L**	−0.29**	−0.12	−0.19**	0.14*	−0.26**	0.08	0.33**	0.24**	0.24**	1		
**I3L**	−0.32**	−0.15*	−0.07	0	−0.24**	0.01	0.26**	0.12	0.54**	0.50**	1	
**Ht**	−0.25**	0.1	0.11	0.20**	−0.24**	0.12	0.35**	0.02	0.1	0.37**	0.35**	1

Q18	HI	GY	GN	BM(A+7d)	SPI	LPI	TSPI	I2PI	I3PI	I2L	I3L	Ht
**BM(A+7d)**	−0.03	0.04	0.03	1								
**LPI**	−0.05	−0.03	−0.06	0.04	−0.16*	1						
**TSPI**	−0.32**	−0.12	−0.11	−0.15*	−0.39**	0.17*	1					
**I2PI**	−0.18*	−0.08	−0.01	−0.11	−0.34**	0.20*	0.41**	1				
**I3PI**	−0.51**	−0.1	−0.14*	0.07	−0.21**	0.06	0.28**	0.59**	1			
**I2L**	−0.40**	−0.12	−0.12	0.02	−0.46**	0.11	0.46**	0.25**	0.17*	1		
**I3L**	−0.31**	−0.21**	−0.18*	−0.01	−0.29**	−0.05	0.31**	0.21*	0.25**	0.64**	1	
**Ht**	−0.24**	−0.1	−0.05	−0.02	−0.13	−0.05	−0.05	0.08	0.14	0.33**	0.37**	1

Combined	HI	GY	GN	BM(A+7d)	SPI	LPI	TSPI	I2PI	I3PI	I2L	I3L	Ht
**BM(A+7d)**	0.03	0.12	0.13	1								
**LPI**	−0.16*	−0.04	−0.04	0.17*	−0.25**	1						
**TSPI**	−0.13*	−0.06	−0.08	0.05	−0.65**	−0.07	1					
**I2PI**	−0.21**	−0.12	−0.13*	−0.12	−0.31**	0.04	0.28**	1				
**I3PI**	−0.26**	−0.21**	−0.21**	−0.06	−0.25**	0.01	0.28**	0.70**	1			
**I2L**	−0.17*	0.06	0.001	0.14*	−0.25**	0.09	0.25**	0.24**	0.13	1		
**I3L**	−0.27**	−0.17**	−0.21**	0.10	−0.38**	0.15*	0.31**	0.13	0.25**	0.30**	1	
**Ht**	0	0.12	0.02	0.20**	−0.29**	0.04	0.32**	−0.06	−0.01	0.18**	0.24**	1

*Note*: C18, Q17, Q18, and Combined refers to Citra 2018, Quincy 2017, Quincy 2018, and combined datasets, respectively.

Abbreviations: BM (A+7d), above‐ground biomass at anthesis+7 days in g m^−2^; GN, grain number m^−2^; GY, grain yield in kg ha^−1^; HI, harvest index; Ht, plant height in cm; I2L, internode 2 length in cm; I2PI, internode 2 partitioning index; I3L, internode 3 length in cm; I3PI, internode 3 partitioning index; LPI, lamina partitioning index; SPI, spike partitioning index; TSPI, true stem partitioning index.

***, **, and * denote significance at 0.001, 0.01, and 0.05 probability levels, respectively.

**TABLE 3:** Direct and indirect effects of different traits on harvest index (HI) identified through path coefficient analysis in combined dataset.

Traits	Direct effect	GN	BM(A+7d)	SPI	LPI	TSPI	I2PI	I3PI	I2L	I3L	Ht
GN	0.391**	0.391	0.004	0.024	−0.017	0.001	−0.001	0.012	0.000	0.005	0.001
BM(A+7d)	0.024	0.070	0.024	−0.003	−0.021	−0.001	−0.001	0.007	−0.001	−0.013	−0.011
SPI	0.102*	0.092	−0.001	0.102	0.014	0.010	−0.001	0.017	0.000	0.034	0.014
LPI	−0.125*	0.052	0.004	−0.011	−0.125	0.001	0.000	−0.001	0.000	−0.019	−0.002
TSPI	−0.02	−0.016	0.001	−0.051	0.008	−0.020	0.002	−0.030	−0.001	−0.038	−0.018
I2PI	0.006	−0.057	−0.003	−0.023	−0.005	−0.006	0.006	−0.075	−0.001	−0.015	0.004
I3PI	−0.107	−0.044	−0.001	−0.016	−0.001	−0.006	0.004	−0.107	−0.001	−0.031	0.000
I2L	−0.004	0.015	0.003	−0.012	−0.011	−0.005	0.001	−0.014	−0.004	−0.037	−0.010
I3L	−0.122*	−0.017	0.002	−0.029	−0.019	−0.006	0.001	−0.027	−0.001	−0.122	−0.014
Ht	−0.057	−0.006	0.005	−0.025	−0.005	−0.006	0.000	0.001	−0.001	−0.029	−0.057

Abbreviations: BM (A+7d), above‐ground biomass at antheisis+7 days; GN, grain number; SPI, spike partitioning index; Ht, plant height; I2L, internode 2 length; I2PI, internode 2 partitioning index; I3L, internode 3 length; I3PI, internode 3 partitioning index; LPI, lamina partitioning index; TSPI, true stem partitioning index.

***, **, and * denote significance at 0.001, 0.01, and 0.05 probability levels, respectively.

### 3.3 PC biplot analysis

**FIGURE 1:**
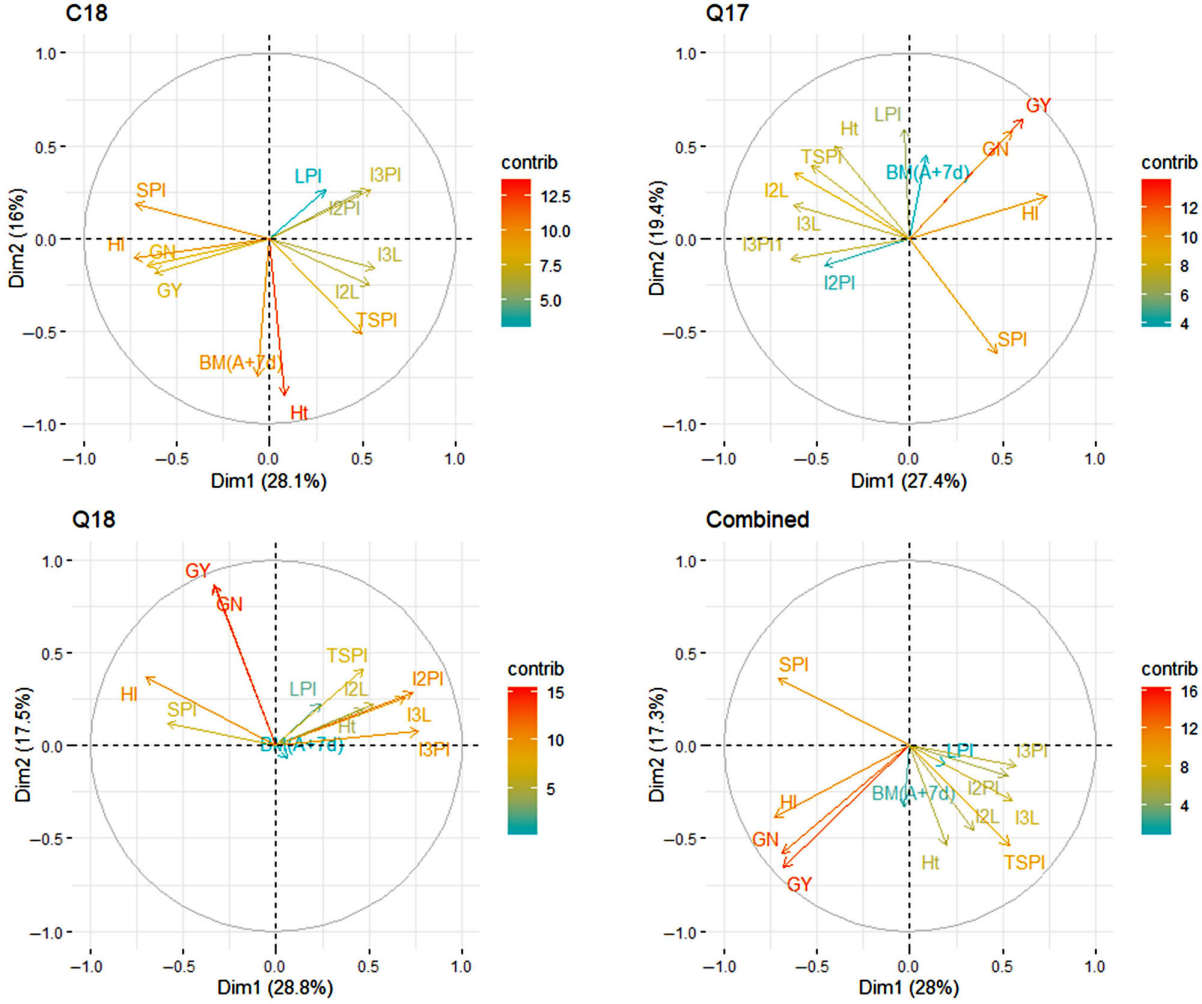
Principal component biplot analysis of measured traits using best linear unbiased estimates (BLUEs). BM (A+7d), above ground biomass at antheisis+7 days in g m^−2^; GN, grain number m^−2^; GY, grain yield in kg ha^−1^; HI, harvest index; Ht, plant height in cm; I2L, internode 2 length in cm; I2PI, internode 2 partitioning index; I3L, internode 3 length in cm; I3PI, internode 3 partitioning index; LPI, lamina partitioning index; SPI, spike partitioning index; TSPI, true stem partitioning index.

The result obtained from correlation analysis was further supported by PC biplot analysis. The analysis showed the association between HI and other traits based on the correlation matrix. The first PCs explained 27.4% (Q17) to 28.8% (Q18), whereas the second PCs explained 16% (C18) to 19.4% (Q17) of the variation (Figure 1). In the PC biplot, HI showed a close association with GY, GN, and SPI, whereas TSPI, I2PI, and I3PI were clustered together in a different group on a negative axis (Figure 1).

### 3.4 Genetic data, population structure, and LD decay

After filtering for MAF and missing data, we obtained 20,706 SNPs with 7935 (38.32%) on A, 7496 (36.20%) on B, and 5275 (25.48%) on D genomes (Figure S2). The SNP markers are distributed throughout the 21 chromosomes. The highest number of SNPs was found on chromosome 3B (1338 SNPs), whereas the lowest was on chromosome 4D (456 SNPs). The total physical map covered 13,800 Mb with an average marker density of 0.67 Mb per marker (Figure S3).

**FIGURE 2:**
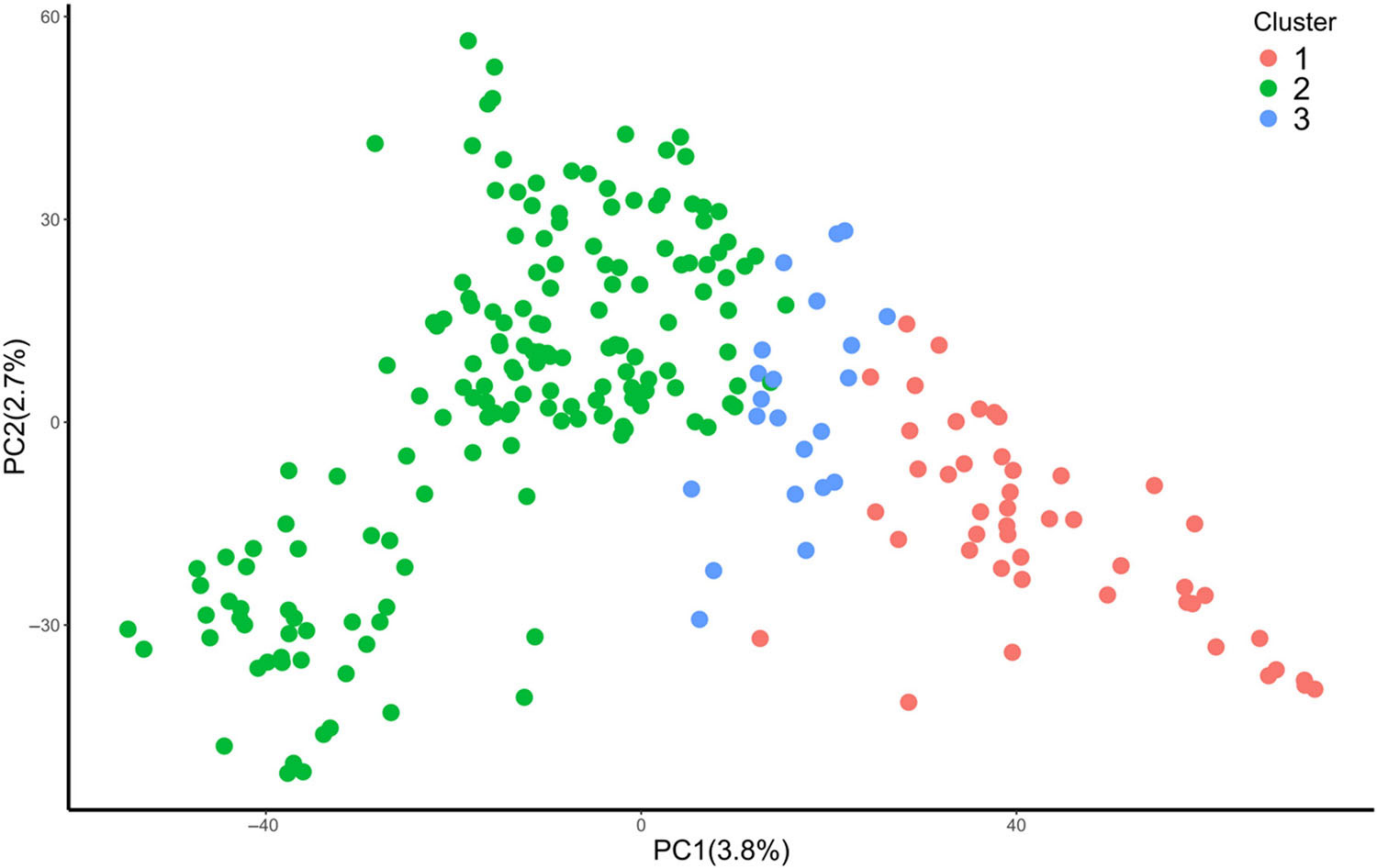
Population structure of genotypes based on 20,706 single nucleotide polymorphisms (SNPs) among groups inferred from principal component (PC) analysis.

The DAPC analysis suggested three genetic groups (clusters) and showed admixture among genotypes in the association mapping panel. The first and second PCs explained 3.8% and 2.7% of the total genotypic variance, respectively (Figure 2). LD decay varied among genomes. The LD decay line below the line of critical value (*r* ^2 ^= 0.1), beyond which we observed that LD was due to linkage, was estimated at 1.8 Mbp (Megabase pair), 12.5 Mbp, and 1.06 Mbp for A, B, and D genome, respectively. In this panel, the LD decay was about 3.4 Mbp for the whole genome (Figure S4).

### 3.5 Marker‐trait association

**FIGURE 3:**
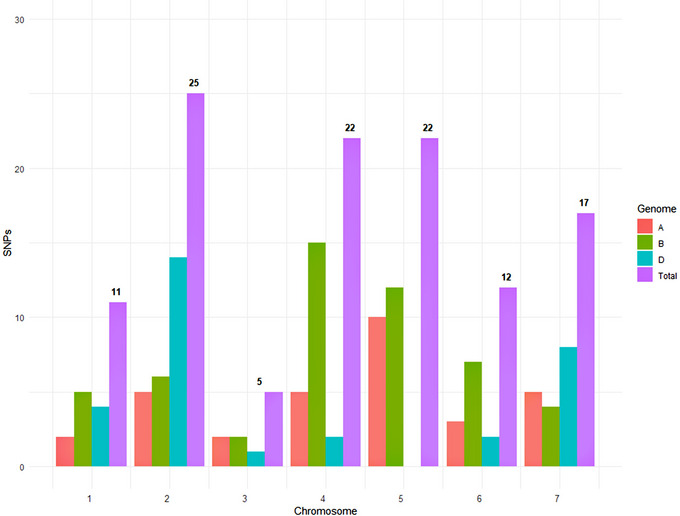
Marker‐trait associations across three genomes in US soft wheat association panel. SNPs, single nucleotide polymorphisms.

For the 12 traits that we studied, GWAS revealed 114 unique significant MTAs across 20 chromosomes, which explained 7.7%–36.62% of the variation (phenotypic variation explained [PVE]) for different MTAs (Tables S3 andS4). Chromosome 4B showed the highest number of significant SNPs (15) followed by 2D (14) and 5B (12) (Figure 3). The lowest number of significant SNPs (one) was found in chromosome 3D. Of the 114 MTAs, 32 MTAs were detected on A genome, 51 MTAs on B genome, and 31 MTAs on D genome. The highest number of MTAs were observed for LPI (28) followed by I3L (25), whereas HI had the fewest significant SNPs (two) (Table S3).

**FIGURE 4:**
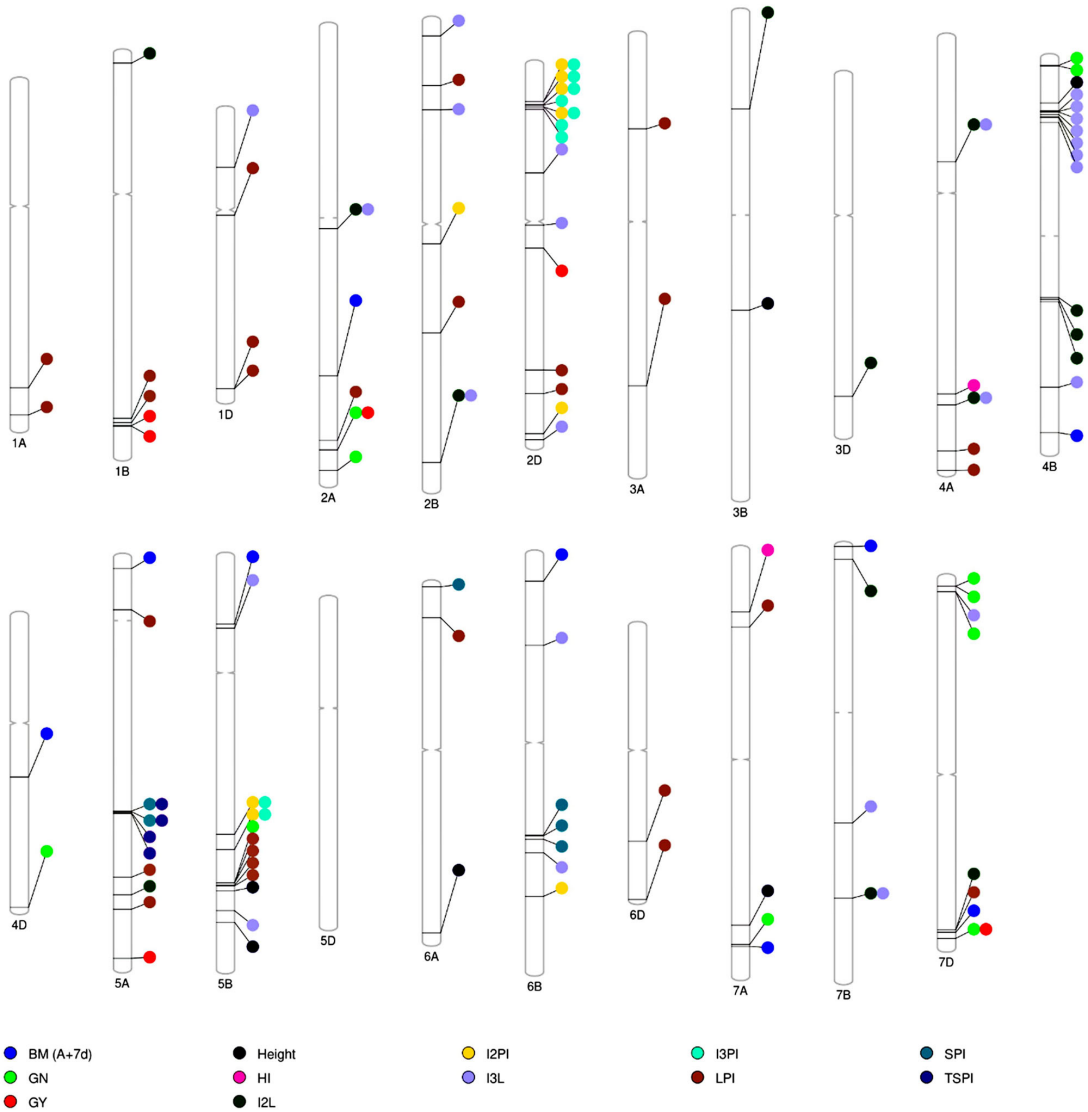
Phenogram showing significant markers trait associations identified on each chromosome for 12 phenotypic traits obtained from the genome‐wide association study (GWAS). BM (A+7d), above‐ground biomass at anthesis+7 days in g m^−2^; GN, grain number m^−2^; GY, grain yield in kg ha^−1^; HI, harvest index; Ht, plant height in cm; I2L, internode 2 length in cm; I2PI, internode 2 partitioning index; I3L, internode 3 length in cm; I3PI, internode 3 partitioning index; SPI, spike partitioning index; LPI, lamina partitioning index; TSPI, true stem partitioning index.

The phenogram (Figure 4) represents the genome‐wide distribution of significant MTAs on specific chromosomes for each trait. The GWAS identified two MTAs (∼16% PVE) for HI located on chromosomes 4A and 7A (Table S3, Figure 4, Figure S5a), and six MTAs for GY on chromosomes 1B, 2A, 2D, 5A, and 7D that explained 10.78%–15.61% of variation (Table S3, Figure 4, Figure S5b). Eleven significant MTAs for GN were identified on chromosomes 2A, 4B, 4D, 5B, 7A, and 7D with single loci explaining 9.27%–16.76% of the phenotypic variance (Table S3, Figure 4, Figure S5c). Nine MTAs were identified for BM(A+7d) on chromosomes 2A, 4D, 5A, 5B, 6B, 7A, 7B, and 7D explaining 9.89%–20.51% of the phenotypic variation (Table S3, Figure 4, Figure S5d). For SPI, six significant MTAs were detected on chromosomes 5A, 6A, and 6B that explained 9.42%–13.54% of the phenotypic variation (Table S3, Figure 4, Figure S5e). A total of 28 SNPs on 14 chromosomes were significantly associated with LPI and explained 8.17%–36.62% of the phenotypic variation (Table S3, Figure 4, Figure S5f). Four significant MTAs for TSPI were located on chromosome 5A with PVE ranging from 9.05% to 10.37% (Table S3, Figure 4, Figure S5g). Fourteen significant SNPs for I2L were detected on 10 chromosomes with 8.53%–17% PVE (Table S3, Figure 4, Figure S5h). We found 25 MTAs for I3L on 10 different chromosomes, which individually explained 7.7%–16.65% of the variation (Table S3, Figure 4, Figure S5i). I2PI was associated with nine significant markers on four chromosomes with PVE ranging from 11.47% to 14.89% (Table S3, Figure 4, Figure S5j). Nine significant MTAs for I3PI on chromosomes 2D and 5B were found (Table S3, Figure 4, Figure S5k) and explained phenotypic variability ranging from 9.58% to 19.05% (Table S3). For Ht, six MTAs were detected on chromosomes 3B, 4B, 5B, 6A, and 7A (Table S3, Figure 4, Figure S5l) and explained 9.80%–16.01% of the phenotypic variation (Table S3).

### 3.6 Haplotype block analysis

The haplotype analysis identified 19 haploblocks (possible QTL locations) within the range of 335–100,432 kbp. We found the highest number of haploblocks for LPI (six) (Figure S6i) followed by GN (four) (Figure S6b). Likewise, I2PI (Figure S6f) and SPI (Figure S6c) had two haploblocks, whereas GY (Figure S6a), TSPI (Figure S6d), I2L (Figure S6e), I3PI (Figure S6h), and I3L (Figure S6g) had one haploblock each.

### 3.7 MTAs with pleiotropic effects

Fifteen pleiotropic loci were detected for GY, GN, I2L, I3L, I2PI, I3PI SPI, and TSPI (Table 4). Markers associated with GY on chromosome 2A (S2A_723531989) and 7D (S7D_621775872) showed pleiotropic effects on GN. Likewise, two MTAs on chromosome 5A (S5A_437344490 and S5A_438411966) showed pleiotropy on SPI and TSPI. Traits I2PI and I3PI shared six pleiotropic loci on chromosomes 2D and 5B. Five and two loci had pleiotropic effects on I2L and I3L, and SPI and TSPI, respectively.

**TABLE 4:** Summary of pleiotropic markers and genes associated with different traits in association panel.

Traits	SNP	Chr.	Position	Alleles	Gene ID	Annotation
I2L, I3L	S2A_345165899	2A	3.45E+08	A/G		
GN, GY	S2A_723531989	2A	7.24E+08	T/C	TraesCS2A02G489700	Carboxypeptidase
I2L I3L	S2B_756106557	2B	7.56E+08	A/T	TraesCS2B02G564200	Haem oxygenase like, multiple helical
I2PI, I3PI	S2D_63397906	2D	63397906	T/G		
I2PI, I3PI	S2D_63397962	2D	63397962	C/T		
I2PI, I3PI	S2D_68734015	2D	68734015	C/G	TraesCS2D02G119100	Nitrate‐inducible and auto‐repressible transcriptional repressor
I2PI, I3PI	S2D_72613962	2D	72613962	A/G		
I2L, I3L	S4A_212894827	4A	2.13E+08	C/T		
I2L,I3L	S4A_628621811	4A	6.29E+08	G/A	TraesCS4A02G353700	
SPI, TSPI	S5A_437344490	5A	4.37E+08	T/C		
SPI, TSPI	S5A_438411966	5A	4.38E+08	T/C	TraesCS5A02G222300	Leucine‐rich repeat, typical subtype
I2PI, I3PI	S5B_479718074	5B	4.8E+08	C/A	TraesCS5B02G296400	Dolichol‐phosphate mannosyltransferase subunit 3
I2PI, I3PI	S5B_506319187	5B	5.06E+08	C/T	TraesCS5B02G320800	Homeobox‐like domain superfamily
I2L, I3L	S7B_608375522	7B	6.08E+08	A/G	TraesCS7B02G351400	Prolamin‐like domain
GN, GY	S7D_621775872	7D	6.22E+08	C/T		

Abbreviations: GN, grain number m^−2^; GY, grain yield in kg ha^−1^; SPI, spike partitioning index; I2L, internode 2 length in cm; I2PI, internode 2 partitioning index; I3L, internode 3 length in cm; I3PI, internode 3 partitioning index; TSPI, true stem partitioning index.

### 3.8 Gene annotation

Functional gene annotation of all significant MTAs was carried out using IWGSC v1.0 sequence assembly. Fifty‐eight out of 114 unique MTAs were anchored within genes with a wide range of biological and metabolic functions (Table S4). Likewise, five genes that are in proximity (∼200 kb) of significant SNPs were also found (Table S5). Candidate genes associated with SNPs were investigated for their functions with reference to past literature. We identified putative candidate genes encoding different classes of proteins that have suggestive roles in response to biotic, abiotic, and metabolic traits including flavoprotein, F‐box family protein, zinc finger, P‐loop containing nucleoside triphosphate hydrolase, protein kinase, and so on.

### 3.9 KASP marker validation

**FIGURE 5:**
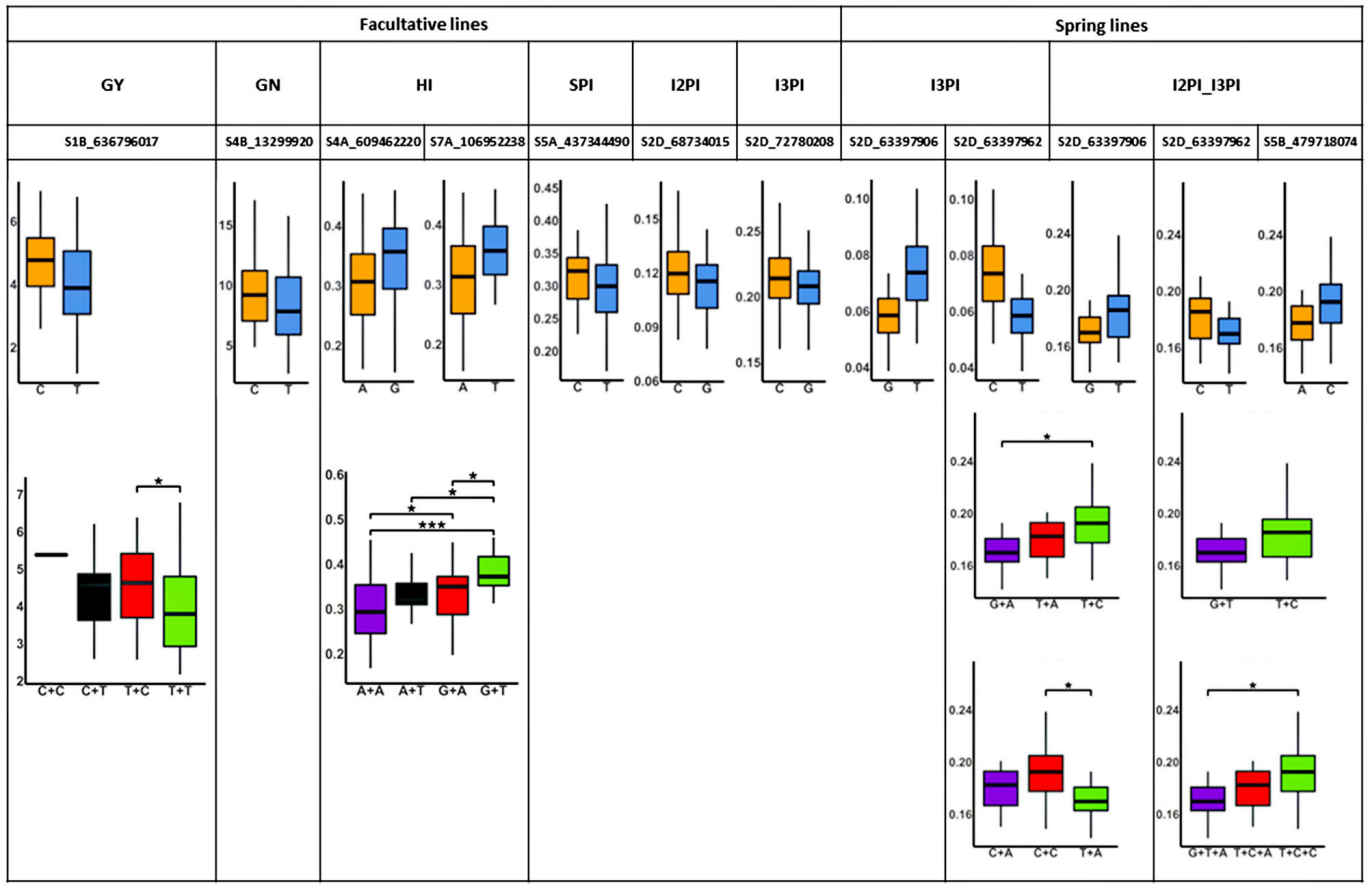
Boxplot showing kompetitive allele specific PCR (KASP) markers that had significant difference in trait values between bi‐ and multi‐allelic groups in the diversity population. The KASP markers were identified using a studentized *t*‐test at *p*‐value < 0.05 in each panel (spring and facultative wheat populations). GN, grain number m^−2^; GY, grain yield in kg ha^−1^; HI, harvest index; I2PI, internode 2 partitioning index; I2PI_I3PI, internode 2 and 3 partitioning index; I3PI, internode 3 partitioning index; SPI, spike partitioning index.

Out of 49 KASP markers developed, a total of 12 trait associated KASP markers were significantly associated with GY, GN, HI, SPI, I2PI, and I3PI traits in the two validation populations (Figure 5, Table S6). Among them, eight KASP markers were significant for GY, HI, I2PI, and I3PI. The Variant Effect Predictor search in Ensembl (http://plants.ensembl.org/index.html) showed that four KASP marker are associated with identified protein sequences including *C2H2*‐type domain‐containing protein, N‐acetyltransferase domain‐containing protein, *HTH* myb‐type domain‐containing protein, and Dolichol‐phosphate mannosyl‐transferase subunit 3. Several KASP makers also showed multi‐trait mean difference on other traits. For HI, both markers were highly contributing to the yield traits such as GY and GN. For internode partitioning traits, they were highly associated with each other, as well as GN. There were also multi‐allele type of KASP marker effects observed in the validation population.

## 4 DISCUSSION

HI is a complex quantitative trait that enhances GY potential in wheat. There has been no significant progress in the HI of wheat in recent decades (Pradhan et al., 2019; M. Reynolds et al., 2009). Several studies indicate that genetic increases in GY in modern wheat cultivars in recent decades have been associated with increased biomass and grain weight without much gain in HI (Aisawi et al., 2015; Lopes & Reynolds, 2012; Shearman et al., 2005). Scientists predict that still more biomasses can be achieved by increasing radiation use efficiency (M. Reynolds et al., 2012). To exploit current and future improvement in biomass production for increased GY, HI also needs to be increased (Lopes & Reynolds, 2012; Rivera‐Amado et al., 2019; Sierra‐Gonzalez et al., 2021). This could be achieved by identifying traits and associated markers that enable discrimination between “useful” and “non‐useful” biomass to improve partitioning toward the grain. Various research has shown that GN plays an important role in improving HI and GY (Foulkes et al., 2011; Pradhan et al., 2019). Likewise, SPI showed a significantly positive correlation with HI and GN (Ferrante et al., 2010, 2013; M. Reynolds et al., 2012; Rivera‐Amado et al., 2019; Shahi et al., 2022; Slafer et al., 2015). Although HI is mostly negatively correlated with biomass at maturity, we found HI had no significant association with BM(A+7d), indicating that genotypes depend more on SPI rather than biomass at anthesis to increase HI. Additionally, there was a very strong negative significant association between SPI and TSPI, implying strong competition between spike and true stem, that is, high DM partitioning to the stem resulting reduced assimilate partitioning to the spike. We also found that the relationship between internodes and SPI differs based on the position of the internodes. SPI had negative significant associations with I2PI, I3PI, I2L, and I3L. It points to the competition between spike and internode 2 and internode 3 for assimilates during the stem elongation stage when fertile florets and GNs are determined. Our study also found a lack of significant association of SPI with internode 1 (peduncle) length and DM partitioning (data nor presented), consistent with a recent study (Rivera‐Amado et al., 2019). It strengthens the hypothesis that peduncle elongation and its DM accumulation occurs after anthesis, whereas the second and the third internode growth coincide with the rapid spike growth phase, which happens before anthesis (Rivera‐Amado et al., 2019; Sierra‐Gonzalez et al., 2021). Overall, SPI had a significant positive association with HI, whereas TSPI, I2PI, I3PI, I2L, and I3L had a significant negative association with HI. Path coefficient analysis also displayed negative direct effects of stem partitioning traits like I2PI, I3PI, I2L, and I3L on HI. Since internode 2 and 3 had been identified as part of the true stem that competes most strongly with spike growth during stem elongation and had a negative impact on HI, the reduction of their lengths and weights might result in increased SPI, and thus would result in improved HI. Furthermore, the structural component of wheat stem needs to be targeted since the non‐soluble stem component is known to compete strongly with spike/floret during the pre‐anthesis rapid spike growth phase while also considering the maintenance of morphological integrity of plant so that there is no lodging. LPI had a negative correlation with SPI, whereas it had a nonsignificant association with HI. Although leaf lamina also competes with spike, reducing lamina biomass might result in photosynthetic costs and it is also important to note that Austin (1980), when he estimated the upper theoretical limit of HI to be 0.62, did not consider reducing leaf lamina biomass to maximize HI. Plant height showed a significant negative correlation with HI and a significant positive correlation with BM(A+7d) and GY, which was similar to previous studies (Aisawi et al., 2015; Fischer, 2007). Plant height also showed a significant negative correlation with SPI in agreement with a previous study (Aisawi et al., 2015). There was no significant association with GN and Ht, although various studies have shown that the reduction in plant height led to improved grains per square meter (Aisawi et al., 2015; Fischer, 2007). However, there is a limitation to plant height manipulation since a further reduction in plant height would lead to biomass penalties and a further increase could decrease lodging resistance. Therefore, for future GY increase, it will be critical to identify strategies for increasing grain sink while maintaining plant height (Foulkes et al., 2009, 2011; M. Reynolds et al., 2012). We found a significant difference for the partitioning traits (SPI, TSPI, LPI, I2L, I3L, I2PI, and I3PI) that indicates the presence of diversity and scope for selection of these traits for enhancement of HI and GY in wheat. These findings are also supported by another associated study in CIMMYT high biomass containing spring wheat lines (Lázaro & Abbate, 2012; Rivera‐Amado et al., 2019). The heritability of major portioning traits was low to medium. This indicates high environmental influence and complex nature of these traits. To improve the estimation of heritability of these traits for future studies, there is a need to involve more replications, more uniform environments, and accurate measurement by reducing human errors.

GWAS is a very useful tool for the genetic dissection of quantitative traits and understanding these traits at the nucleotide level. GWAS identified 114 unique significant MTAs for various traits in the present study. Our study revealed pleiotropic markers that control GY and GN, as well as SPI and TSPI. Traits like I2PI, I3PI, I2L, I3L, and TSPI had a negative effect on HI. To raise the yield potential in the future, wheat needs to reduce extra assimilates partitioning toward the structural stem component (without compromising biological process and structural strength) and invest them toward producing grains. Increasing both sink size and capacity by adjusting and tweaking different partitioning traits (spike partitioning, internode partitioning, etc.) with the help of marker‐assisted selection tools provides us with a great opportunity to increase HI in wheat.

For HI, two significant MTAs were found on chromosomes 4A and 7A (Table S4). Previous reports identified MTAs for HI on wheat chromosome 2A (Bhatta et al., 2018), 3A (Bhatta et al., 2018; Pradhan et al., 2019), 3B (Sukumaran et al., 2018), 3D (Bhatta et al., 2018), 5B (Bhatta et al., 2018; Pradhan et al., 2019), and 6B (Bhatta et al., 2018). Out of the six MTAs identified for GY, two MTAs were found within genes (Table S4). Previous studies reported that QTLs/MTAs are responsible for GY on 1B (Bhatta et al., 2019; Edae et al., 2014; Jamil et al., 2019; F. Li et al., 2019), 2A (Jamil et al., 2019; F. Li et al., 2019; Pradhan et al., 2019), and 2D (Edae et al., 2014; Jamil et al., 2019; F. Li et al., 2019). An MTA S1B_636796017 associated with GY was found in the gene *TraesCS1B02G409500* and this gene codes a C_2_H_2_‐type zinc finger family protein (Table S4) that promotes plant growth, development, and stress signal transduction (Han et al., 2020). It has also been found to provide abiotic stress tolerance and enhance GN in cereal crops such as rice (Giri et al., 2011; Han et al., 2020). For GN, five out of 11 significant MTAs were found within different genes (Table S4). The significant MTA S4B_12533949 was found in the gene *TraesCS4B02G017400* on the chromosome 4B (Table S4) and is annotated as an inositol polyphosphate‐related phosphatase responsible for stress tolerance (Jia et al., 2019). The gene *TraesCS4B02G018300* associated with an MTA S4B_13299920 encodes acyl‐CoA N‐acyltransferase proteins with functions such as embryo and seed development, seed dormancy and germination, seedling development, reproductive growth, plant senescence, and tolerance to various environmental stresses (Du et al., 2016; Walla et al., 2020). Four MTAs were found within genes for BM (A + 7d). An MTA S6B_4578033 is located on gene TraesCS6B02G0678000 that encodes flavoprotein. Flavoproteins are involved in the production of the hormone auxin (IAA) in Arabidopsis, an essential hormone for the biological processes such as plant growth, regulating embryogenesis, as well as leaf, root, flower, vascular and fruit development, and stress responses such as drought tolerance (Lee et al., 2012; Schall et al., 2020; Thodberg & Neilson, 2020).

Our study identified six MTAs responsible for SPI located within genes (Table S4). An MTA S5A_438411966 was found on chromosome 5A located within the gene *TraesCS5A02G222300* that codes a leucine‐rich repeat protein (Table S4) regulating signal transduction, pollen tube growth, root development, and defense response to different diseases like powdery mildew in wheat and acting in multiple developmental, environmental, and defense‐related pathways (Acevedo et al., 2004; Suzuki et al., 2004; X. Wang et al., 2016). Thirteen MTAs for LPI were found within genes (Table S4). The MTA S1B_631715420 was found in *TraesCS1B02G401200* annotated as an F‐box family protein (Table S4). These proteins help in the developmental and physiological processes such as spike development, plant hormone response pathways, light signaling, and pollen recognition in plants (Hong et al., 2012; Lechner et al., 2006).

Two MTAs for TSPI were found within genes (Table S4). An MTA on chromosome 5A (S5A_441478636) is in gene *TraesCS5A02G225700* (Table S4) annotated as helix‐loop DNA binding protein. Nine MTAs for I2L were found within genes (Table S4). Out of 25 MTAs for I3L, nine MTAs were found in genes (Table S4). Significant MTA (S4B_101301774) for I3L was located within *TraesCS4B02G097000* (Table S4) on chromosome 4B that is annotated as an Fe‐S cluster assembly domain superfamily protein. This protein has been reported to have a wide range of functions such as electron transfer, metabolic and biosynthetic reactions, and gene expression regulation in mitochondria and chloroplast (Couturier et al., 2013; Lu, 2018). An MTA (S2B 756106557) was located within *TraesCS2B02G564200* on chromosome 2B and the gene encodes Haem oxygenase family protein that is involved in different cellular processes such as cell protection, iron mobilization, phytochrome chromophore synthesis, and stomatal regulation (Shekhawat & Verma, 2010). Likewise, four MTAs for I2PI were found significant (Table S4). The MTA S2D_68734015 was found in *TraesCS2D02G119100* (Table S4) that encodes a nitrate‐inducible and auto‐repressible transcriptional repressor (nitrogen response) protein to guide nitrogen response in rice (Sawaki et al., 2013). GWAS identified nine MTA for I3PI, and five of them were within genes (Table S4). One MTA (S5B_506319187) is located in *TraesCS5B02G32080* encoding a homeobox‐like domain protein. This protein helps plant growth and development and is involved in various hormone response pathways (Li et al., 2017). Lastly, four of the six MTAs for Ht were within genes (Table S4). Since no previous research has been reported on the genes controlling the partitioning traits, these genes we discussed in this paper could be further studied and manipulated for the genetic improvement of wheat. Since most of the partitioning traits studied in this research are laborious and time‐consuming as well as destructive in terms of direct phenotyping them, there is a need to develop strategy to select them indirectly in a breeder‐friendly fashion.

We also validated the significant MTAs identified in GWAS in a different panel of both facultative and spring wheat populations. We identified KASP markers significantly associated with GY and GY‐related partitioning traits such as HI, SPI, and sectional internode weight. This result provides insight into the efficacy and transferability of MTAs on different wheat breeding populations. Only a few studies have validated KASP markers related to yield component traits in wheat. R. Wang et al. (2018) found that three out of eight KASP markers showed significant allelic effect for tillers per unit area and spikelets per spike in both diversity and recombinant wheat inbred lines. We found eight out of 12 KASP markers tested were effective in different panels. Similar to their study, we found trade‐off patterns for certain traits. For example, the KASP marker for I2PI (S2D_68734015) showed a positive effect on GN, but negative effect for I3L. However, the majority of the KASP markers showed synergetic mean difference on yield component traits. The multi‐allele types of KASP marker effect indicate that different strategies should be used for marker‐assisted selection on populations carrying different allele combinations. Using this KASP marker panel, elite lines carrying several benefitting alleles could be selected in breeding programs.

## 5 CONCLUSION

The study demonstrated genetic variations for SPI, LPI, TSPI, and several other stem partitioning traits in US soft wheat. The results provided insight into how to increase HI without compromising critical traits involved in photosynthesis and assimilate transfer from stem and leaf. SPI demonstrated a positive effect on HI and GY by increasing GN. SPI also showed a negative correlation with I2L and I3L. These results suggest that genotypes with decreased second and third internode lengths compete less with the spike for assimilating at anthesis + 7 days and may increase HI and GN. Contrary to that, longer second and third internodes compete more with the spike for assimilating at anthesis+7 days and may decrease SPI, GN, and HI. Hence, SPI, along with other stem partitioning traits I2PI and I3PI, is promising for altering HI, GY, and GN, which could further be used for selection in the wheat breeding programs. The GWAS identified 114 unique significant MTAs for different traits. Among them, 58 MTAs were found within genes and these genes have been associated with important biological processes in plants. MTAs identified from GWAS were successfully converted to KASP marker and most of them were validated in a different population. These validated KASP markers could be used in pyramiding of favorable alleles, selection of promising lines for crossing, and marker‐assisted selection for the genetic improvement of HI and GY potential.

## AUTHOR CONTRIBUTIONS

**Dipendra Shahi**: Data curation; formal analysis; investigation; writing—original draft; writing—review and editing. **Jia Guo**: Data curation; formal analysis; investigation; validation; writing—original draft; writing—review and editing. **Md Ali Babar**: Conceptualization; funding acquisition; investigation; methodology; project administration; resources; supervision; writing—review and editing. **Sumit Pradhan**: Data curation; writing—review and editing. **Muhsin Avci**: Data curation; writing—review and editing. **Naeem Khan**: Data curation; formal analysis; writing—review and editing. **Jordan McBreen**: Data curation; writing—review and editing. **Smita Rayamajhi**: Data curation. **Zhao Liu**: Data curation; formal analysis; methodology; writing—review and editing. **Guihua Bai**: Data curation; formal analysis; funding acquisition; investigation; writing—review and editing. **Paul St. Amand**: Data curation; formal analysis; methodology; writing—review and editing. **Amy Bernardo**: Data curation; formal analysis; investigation; methodology; writing—review and editing. **Mathew Reynolds**: Conceptualization; funding acquisition; investigation; project administration; resources; supervision; writing—review and editing. **Gemma Molero**: Conceptualization; funding acquisition; investigation; resources; supervision; writing—review and editing. **Sivakumar Sukumaran**: Conceptualization; funding acquisition; investigation; methodology; resources; writing—review and editing. **John Michael Foulkes**: Conceptualization; funding acquisition; investigation; methodology; resources; writing—review and editing. **Jahangir Khan**: Data curation; formal analysis; writing—review and editing.

## CONFLICT OF INTEREST STATEMENT

The authors declare no conflicts of interest.

## Supporting information



Supplementary Material

Supplementary Material

## Data Availability

The data and code used for this study can be found at https://github.com/Dipendra031/gwas_study_wheat.
