# Supplementary material for: Deciphering the genetic basis of novel traits that discriminate useful and non‐useful biomass to enhance harvest index in wheat
Source: Plant Genome. 2024 Sep 18;17(4):e20512. doi: 10.1002/tpg2.20512 (PMC11628932; doi:10.1002/tpg2.20512)
Supplement: Supplementary file 1 [file TPG2-17-e20512-s001.docx]

Figure S1. Figure is showing T_ave_ (monthly average temperature in ^o^C) and Ppt (monthly precipitation in mm). Association mapping panel was planted in Citra (2017-2018, 2018-2019) and Quincy (2016-2017, 2017-2018).

Figure S2. Genome wide distribution of 20,706 single nucleotide polymorphisms (SNPs) markers in soft wheat association panel.

**The number of SNPs within 1 MB window size**

Figure S3. SNP Density plot of 20,706 single nucleotide polymorphisms (SNPs) markers

Figure S4. Linkage disequilibrium (LD) represented by the r^2^ against physical distance (Mbp). The dotted line represents the critical value beyond which LD is likely caused by physical linkage.

Figure S5a. Manhattan plots(left) and quantile-quantile plots (right) showing genome-wide SNP loci associated with a) harvest index (HI) ordered on Q17, Q18, C18,and Combined. The horizontal line in Manhattan plot represents the expected value with a uniform suggestive genome wide significance threshold [-FDR ≤ 0.10].

Figure S5b. Manhattan plots(left) and quantile-quantile plots (right) showing genome-wide SNP loci associated with b) grain yield (GY) ordered on Q17, Q18, C18,and Combined. The horizontal line in Manhattan plot represents the expected value with a uniform suggestive genome wide significance threshold [-FDR ≤ 0.10].

Figure S5c. Manhattan plots(left) and quantile-quantile plots (right) showing genome-wide SNP loci associated with c) grain number m^-2^ (GN) ordered on Q17, Q18, C18,and Combined. The horizontal line in Manhattan plot represents the expected value with a uniform suggestive genome wide significance threshold [-FDR ≤ 0.10].

Figure S5d. Manhattan plots (left) and quantile-quantile plots (right) showing genome-wide SNP loci associated with d) above ground biomass at anthesis + 7 days (BM(A+7d) ordered on Q17, Q18, C18,and Combined. The horizontal line in Manhattan plot represents the expected value with a uniform suggestive genome wide significance threshold [-FDR ≤ 0.10].

Figure S5e. Manhattan plots (left) and quantile-quantile plots (right) showing genome-wide SNP loci associated with e) spike partitioning index (SPI) ordered on Q17, Q18, C18,and Combined. The horizontal line in Manhattan plot represents the expected value with a uniform suggestive genome wide significance threshold [-FDR ≤ 0.10].

Figure S5f. Manhattan plots (left) and quantile-quantile plots (right) showing genome-wide SNP loci associated with f) lamina partitioning index (LPI) ordered on Q17, Q18, C18,and Combined. The horizontal line in Manhattan plot represents the expected value with a uniform suggestive genome wide significance threshold [-FDR ≤ 0.10].

Figure S5g. Manhattan plots (left) and quantile-quantile plots (right) showing genome-wide SNP loci associated with g) true stem partitioning index (TSPI) ordered on Q17, Q18, C18,and Combined. The horizontal line in Manhattan plot represents the expected value with a uniform suggestive genome wide significance threshold [-FDR ≤ 0.10].

Figure S5h. Manhattan plots (left) and quantile-quantile plots (right) showing genome-wide SNP loci associated with h) internode 2 length (I2L) The horizontal line in Manhattan plot represents the expected value with a uniform suggestive genome wide significance threshold [-FDR ≤ 0.10].

Figure S5i. Manhattan plots (left) and quantile-quantile plots (right) showing genome-wide SNP loci associated with i) internode 3 length (I3L) The horizontal line in Manhattan plot represents the expected value with a uniform suggestive genome wide significance threshold [-FDR ≤ 0.10].

Figure S5j. Manhattan plot (left) and quantile-quantile plots (right) showing genome-wide SNP loci associated with j) internode 2 partitioning index (I2PI) The horizontal line in Manhattan plot represents the expected value with a uniform suggestive genome wide significance threshold [-FDR ≤ 0.10].

Figure S5k. Manhattan plot (left) and quantile-quantile plots (right) showing genome-wide SNP loci associated with internode 3 partitioning index (I3PI) The horizontal line in Manhattan plot represents the expected value with a uniform suggestive genome wide significance threshold [-FDR ≤ 0.10].

Figure S5l. Manhattan plot (left) and quantile-quantile plots (right) showing genome-wide SNP loci associated with l) plant height (Ht) The horizontal line in Manhattan plot represents the expected value with a uniform suggestive genome wide significance threshold [-FDR ≤ 0.10].

Figure S6a. Haplotype block for grain yield (GY)

Figure S6b. Haplotype block for harvest index (HI)

Figure S6c. Haplotype block for Spike Partitioning Index (SPI)

Figure S6d. Haplotype block for True stem Partitioning Index (TSPI)

Figure S6e. Haplotype block for Internode 2 length (I2L)

Figure S6f. Haplotype block for Internode 2 partitioning index (I2PI)

Figure S6g. Haplotype block for Internode 3 Length (I3L)

Figure S6h. Haplotype block for Internode 3 Partitioning Index (I3PI)

Figure S6i. Haplotype block for Lamina Partitioning Index (LPI)
